# Node Selection and Path Optimization for Passive Target Localization via UAVs

**DOI:** 10.3390/s25030780

**Published:** 2025-01-28

**Authors:** Xiaoyou Xing, Zhiwen Zhong, Xueting Li, Yiyang Yue

**Affiliations:** School of Aeronautics and Astronautics, Sichuan University, Chengdu 610065, China; 2022141510062@stu.scu.edu.cn (X.X.); zhongzhiwen@stu.scu.edu.cn (Z.Z.); 2022141510057@stu.scu.edu.cn (Y.Y.)

**Keywords:** passive target localization, unmanned aerial vehicles (UAVs), Cramer–Rao lower bound (CRLB), node selection method, path optimization method, particle swarm algorithm (PSO)

## Abstract

The performance of passive target localization is affected by the positions of unmanned aerial vehicles (UAVs) at a large scale. In this paper, to improve resource utilization efficiency and localization accuracy, the node selection problem and the path optimization problem are jointly investigated. Firstly, the target passive localization model is established and the Chan-based time difference of arrival (TDOA) localization method is introduced. Then, the Cramer–Rao lower bound (CRLB) for Chan-TDOA localization is derived, and the problems of node selection and path optimization are formulated. Secondly, a CRLB-based node selection method is proposed to properly divide the UAVs into several groups, localizing different targets, and a CRLB-based path optimization method is proposed to search for the optimal UAV position configuration at each time step. The proposed path optimization method also effectively handles no-fly-zone (NFZ) constraints, ensuring operational safety while maintaining optimal target tracking performance. Also, to improve the efficiency of path optimization, particle swarm algorithm (PSO) is applied to accelerate the searching process. Finally, numerical simulations are performed to verify the validity and effectiveness of the proposed methods in this paper.

## 1. Introduction

Unmanned Aerial Vehicle swarms (UAVs) have indeed become integral components of modern military operations, particularly in real-time aerial reconnaissance, surveillance, and various tactical applications. The versatility and technological capabilities provide numerous advantages, and their adoption has significantly transformed the landscape of modern warfare [1,2]. Recently, UAVs have garnered significant attention for their enhanced capabilities in complex missions such as target tracking, localization, and detection [3,4,5]. The distributed nature of UAVs provides robust and flexible operations, particularly in challenging tactical environments.

Generally, UAVs can be burdened by active systems or passive systems to fulfill their tasks. For active localization systems, the locator (such as a radar) actively emits signals to detect and determine the position of targets. While this can provide real-time tracking and high precision, it exposes the location of the locator itself to enemy detection. This makes active localization vulnerable in hostile environments, especially in situations where stealth and secrecy are crucial. Additionally, active localization systems tend to require higher energy consumption and more complex infrastructure, which can be a logistical challenge [6,7]. On the other hand, a passive localization system does not involve emitting signals. Instead, it works by detecting and analyzing the electromagnetic waves emitted by the target’s own electronic devices. The key advantage is that it does not disclose the location of the system receiving the signals, making it inherently more stealthy and harder to detect [8,9,10]. In this case, UAVs’ passive localization technology offers significant strategic advantages in situations where stealth, resource efficiency, and survivability are crucial. Its ability to detect targets without emitting signals makes it a powerful tool in modern military operations [11,12].

### 1.1. Overview

For multi-target passive localization via UAVs, node selection and path optimization are crucial to enhance the accuracy and efficiency of target localization, since through node selection, the UAVs could be properly divided into several groups to locate different targets, and through path optimization, the UAVs could achieve proper positions at each frame, obtaining appropriate observation angles and high-quality measurements that help realize high localization accuracy. Therefore, dynamic node selection and path optimization could help ensure that UAVs are optimally positioned to minimize errors in localization, improving the ability of the UAVs in locating a target.

From the perspective of node selection for the UAVs, node selection could be implemented via classic greedy algorithms to construct minimum spanning trees (MSTs) to select a set of UAVs that minimizes the total communication or distance between them [13,14]. Task scheduling also needs to select a set of UAV nodes to optimize task scheduling and load balancing in distributed UAV systems [15,16].

More recently, reinforcement learning (RL) approaches have shown promising results in UAV path planning and navigation tasks. Deep reinforcement learning (DRL) methods, particularly algorithms like deep Q-networks (DQNs) and proximal policy optimization (PPO), have been successfully applied to autonomous navigation in complex environments [17]. These methods enable UAVs to learn optimal navigation policies through interactions with the environment without requiring complete prior knowledge of the operational space. Particularly noteworthy is the development of cross-platform deep reinforcement learning approaches for autonomous navigation without global localizing information [18], which have demonstrated remarkable adaptability across different scenarios and vehicle platforms. Such end-to-end learning approaches that combine local sensor information with learned policies have significantly advanced the field of autonomous navigation [19].

Additionally, ant colony optimization (ACO) could be deployed for node selection to determine optimal routes for the communication relay of coverage tasks, ensuring minimal energy expenditure while still fulfilling the mission’s objectives [20,21], and for a UAV network, the central leader node selection is essential for coordinating tasks and managing communications [22].

From the perspective of path optimization for UAVs, the heuristic-based A* algorithm is used for path optimization to efficiently find the shortest path [23]. The genetic algorithm (GA) is introduced to cooperative path planning for UAVs. It uses a population of potential solutions (paths), which evolve over generations according to a fitness function, and the algorithm incorporates crossovers with mutation operations to explore the solution space while the fitness function guides the search toward optimal or near-optimal paths that avoid collisions and optimize mission objectives [24]. The differential evolution (DE) algorithm is well suited for UAV path optimization because of its ability to efficiently search high-dimensional spaces while minimizing time and energy consumption [25]. The bat algorithm adapts well to dynamic environments and is used to optimize UAV trajectories in the presence of changing obstacles or mission requirements [26].

A projection algorithm has been deployed to improve the localization accuracy of UAVs, and the algorithm guides UAVs toward way points that are closer to the target. This approach reduces the computational burden associated with numerical searches by projecting UAVs onto more promising paths based on predictive models, thus improving the efficiency and effectiveness of the path optimization process [27]. Although this algorithm considers the application of target passive localization, it only considers one target.

Concerning existing works, it can be seen that in most works, node selection and path optimization are separately investigated, which restricts performance improvements in localization accuracy for multi-targets. Also, node selection methods in existing works are mainly concerned with task scheduling, resources distribution, and communication link construction, and the path optimization methods in the existing works are mainly concerned with the shortest path searching and obstacle avoidance operations with less resource consumption, which ignore the application multi-target passive localization. Additionally, existing path optimization methods rarely consider the integration of no-fly-zone constraints with localization performance optimization, which is crucial for practical UAV operations in restricted airspace. Furthermore, while reinforcement learning approaches have shown promising results in UAV navigation, they often require extensive training data and computational resources, and their performance in multi-target passive localization applications remains largely unexplored. The existing deep learning models for autonomous navigation typically focus on single-platform scenarios and may not effectively accommodate the complex requirements of coordinated UAVs operations for passive localization.

### 1.2. Original Contributions

Obviously, works that focus on multi-target passive localization while jointly considering the node selection and the path optimization are rare, and these are indeed the main topics of this paper. In this paper, we jointly consider the problem of node scheduling and path optimization, and we propose a CRLB-based node selection method and a CRLB-based path optimization method to improve localization accuracies. The original contributions of this paper are as follows:

(1) We propose a novel joint optimization framework that unifies node selection and path optimization for multi-target passive localization. Unlike existing methods that handle these problems separately, our integrated approach simultaneously optimizes resource allocation and path planning, achieving superior performance in both resource utilization and localization accuracy.

(2) We develop a CRLB-based node selection method that effectively partitions UAVs into optimal groups for different target localization. This method uniquely considers the theoretical lower bound of estimation errors to guide grouping decisions, leading to improved resource efficiency and localization performance.

(3) We design a CRLB-based path optimization method that dynamically determines optimal UAVs positions by minimizing the theoretical estimation error bound. This approach enables more accurate target localization performance compared to traditional fixed-formation methods.

(4) We implement the PSO algorithm for UAV path optimization. This implementation significantly improves computational efficiency while maintaining solution quality compared to several existing optimization methods.

### 1.3. Organization

The main contents of this paper are shown as follows. The problems of node selection and path optimization are formulated in Section 2. The algorithm of the node selection based on CRLB is given in Section 3, and the algorithm of the path optimization based on CRLB is given in Section 4. Numerical simulations are given in Section 5. This paper is concluded in Section 6.

## 2. Problem Formulation

In this section, we present a comprehensive formulation of the multi-target passive localization problem using UAVs based on the Chan-TDOA method. First, we establish the fundamental Chan-TDOA localization model. Then, we derive the CRLB for measurement errors in the Chan-TDOA model to evaluate the theoretical lower bound of the localization performance. Finally, we formulate two key optimization problems: the node selection problem for optimal UAVs grouping and the path optimization problem for determining optimal UAVs positions, which together form a complete framework for multi-target passive localization via UAVs. The mathematical models and theoretical analyses presented in this section serve as the foundation for the algorithms developed in subsequent sections.

### 2.1. Target Localization Model Based on the Chan-TDOA Algorithm

**Figure 1:**
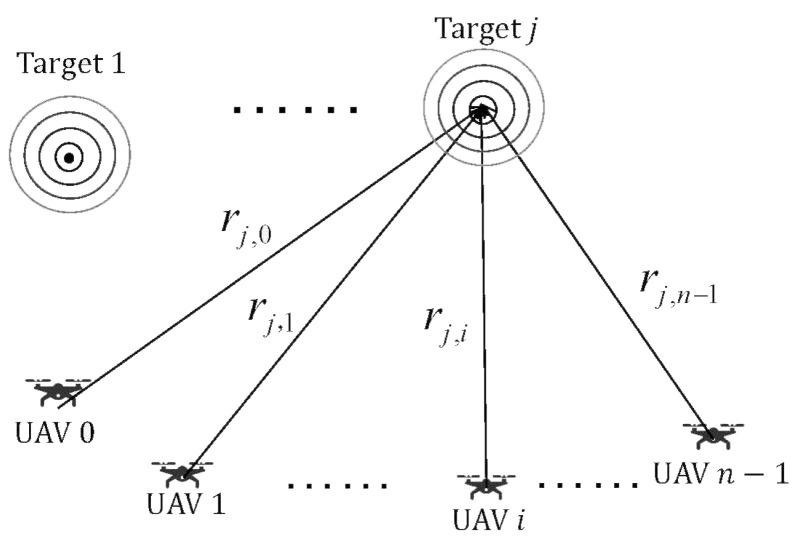
Geometric schematic of the TDOA localization model. The distance between target *j* and each UAV is denoted as $r_{j,i}$, where UAV 0 serves as the reference node (or master UAV), and UAV *i* (i=0,1,…,n−1) represents other nodes in the swarm. The concentric circles around the target indicate the signal propagation.

In sight of the fact that target localization is designed to determine target positions based on measurements, we first establish the fundamental TDOA model. Consider a two-dimensional passive localization scenario with multiple UAVs and targets, as shown in Figure 1.

Assume that there are *m* stationary targets, and the *j*th target has coordinates $w_{j}={\left[x_{j},y_{j}\right]}^{T}$, where j=1,2,…,m. A swarm of *n* UAVs equipped with passive radars is deployed, and their coordinates are given by $u_{i}={\left[x_{i},y_{i}\right]}^{T}$, where i=0,1,…,n−1. Without loss of generality, UAV 0 is selected as the reference node (or master UAV).

The distance between the *i*th UAV and *j*th target can be expressed as

(1) $$r_{j,i}=∥u_{i}-w_{j}∥=\sqrt{{\left(x_{i}-x_{j}\right)}^{2}+{\left(y_{i}-y_{j}\right)}^{2}}.$$

For TDOA measurements, let $\tau_{j,i}$ represent the time taken for the signal from target *j* to reach UAV *i*. The time difference of arrival between UAV *i* and the master UAV (UAV 0) is denoted as $\tau_{j,i,0}$, where the subscript triplet (j,i,0) has the following meaning: The first subscript *j* indicates the target being localized, the second subscript *i* represents the measuring UAV, and the third subscript 0 refers to the master UAV (UAV 0). Thus, $\tau_{j,i,0}=\tau_{j,i}-\tau_{j,0}$ represents the time difference between when UAV *i* and UAV 0 receive the signal from target *j*, which can be written as

(2) $$\tau_{j,i,0}=\tau_{j,i}-\tau_{j,0}=\frac{r_{j,i}-r_{j,0}}{c},$$

where *c* is the propagation speed of electromagnetic waves.

In practical applications, the measured TDOA value $\tau_{j,i,0}^{\prime }$ inevitably contains measurement errors:

(3) $$\tau_{j,i,0}^{\prime }=\tau_{j,i,0}+\Delta \tau_{j,i,0},$$

where $\tau_{j,i,0}^{\prime }$ represents the measured time difference between UAV *i* and UAV 0 for target *j*, $\tau_{j,i,0}$ is the true time difference, and $\Delta \tau_{j,i,0}$ denotes the time difference error, and it is typically modeled as zero-mean Gaussian noise:

(4) $$\Delta \tau_{j,i,0}∼N\left(0,\sigma_{i,j,0}^{2}\right),$$

where $\sigma_{j,i,0}^{2}=\sigma_{j,i}^{2}+\sigma_{j,0}^{2}$ is the variance of the TDOA measurement for target *j*, with $\sigma_{j,i}^{2}$ and $\sigma_{j,0}^{2}$ being the TDOA measurement variances in UAV *i* and UAV 0 respectively.

Several factors contribute to the TDOA measurement errors. The internal electronic noise in the radar receiver affects TDOA measurement precision. Signal reflection and refraction during propagation can introduce additional errors. Variations in atmospheric conditions, such as temperature and humidity, impact the propagation speed. The radar’s time resolution also imposes limitations on measurement capabilities. Also, the standard deviation of the TDOA time measurement $\sigma_{j,i}$ between UAV *i* and target *j* exhibits dependencies on their separation distance:

(5) $$\sigma_{j,i}=\kappa r_{j,i}^{\nu },$$

where κ and ν are system parameters determined by the radar characteristics and propagation environment. The parameter ν is typically close to 1/2, reflecting the relationship between signal power attenuation and distance.

For target localization in two dimensions, at least three UAVs are required to obtain sufficient TDOA measurements. When the number of UAVs n≥3, the TDOA measurement equations can be used to construct a nonlinear system as follows:

(6) $$\left\{\begin{array}{c} c\tau_{j,1,0}^{\prime }=∥u_{1}-w_{j}∥-∥u_{0}-w_{j}∥\\ c\tau_{j,2,0}^{\prime }=∥u_{2}-w_{j}∥-∥u_{0}-w_{j}∥\\ \vdots \\ c\tau_{j,n-1,0}^{\prime }=∥u_{n-1}-w_{j}∥-∥u_{0}-w_{j}∥. \end{array}\right.$$

To accelerate the calculation process of nonlinear equations, the Chan algorithm is introduced in this paper to provide an efficient closed-form solution through linearization and two-step weighted least squares estimation. The Chan algorithm is a non-recursive solver for systems of hyperbolic equations that can transform an initially nonlinear system into a linear one. Under the assumption that the TDOA measurement errors follow an ideal Gaussian distribution, the Chan-TDOA localization algorithm can provide high localization accuracies with reduced computational complexity.

**Figure 2:**
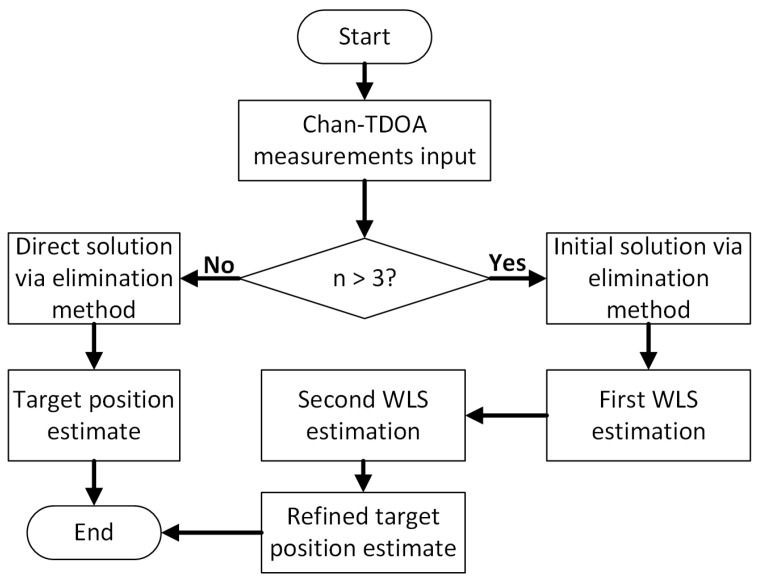
Flow chart of the Chan-TDOA algorithm.

For target localization in two dimensions with multiple UAVs (n≥3), the Chan-TDOA algorithm provides an efficient closed-form solution through linearization and two-step weighted least squares (WLS) estimation. The algorithm first transforms the nonlinear TDOA equations into linear forms through algebraic manipulation. Then, it employs a two-step WLS estimation process to obtain the target location estimate, with the first step providing an initial solution and the second step refining the estimate. The detailed mathematical derivations of the algorithm are presented in Appendix A. The overall flow of the algorithm is illustrated in Figure 2.

### 2.2. CRLB Derivation for TDOA Measurement Errors in the Chan-TDOA Model

The CRLB is an important metric for evaluating parameter estimation performances, representing the smallest possible variance achieved via an unbiased estimator. It is commonly used as a benchmark for comparing the performance of unbiased estimators.

In the context of the Chan-TDOA passive target localization problem discussed in this paper, the observation data of the localization system consists of the TDOA measurements between each UAV and the target. Let the measured TDOA values be denoted as s, with the true values given by

(7) $$s=c{\left[\tau_{1,0},\tau_{2,0},\ldots ,\tau_{n-1,0}\right]}^{T},$$

where $\tau_{i,0}=\tau_{i}-\tau_{0}$ represents the difference in the times at which the signal was received by the *i*th UAV and the master base station. The measurement is given by

(8) $$s^{\prime }=s+m.$$

The TDOA measurement error m follows a normal distribution N(0,Q), where Q is the covariance matrix of the TDOA measurements, as given in (A13). The probability density function of the TDOA measurements can be expressed as

(9) $$p\left(s^{\prime };s\right)=\frac{1}{\sqrt{2\pi |Q|}}\exp\left(-\frac{1}{2}{\left(s^{\prime }-s\right)}^{T}Q^{-1}\left(s^{\prime }-s\right)\right).$$

The likelihood function calculated from this density function is as follows:

(10) $$\ln p\left(s^{\prime };s\right)=-\frac{1}{2}\ln\left|2\pi Q\right|-\frac{1}{2}{\left(s^{\prime }-s\right)}^{T}Q^{-1}\left(s^{\prime }-s\right).$$

Taking the second partial derivatives of (10) with respect to the position’s coordinate parameters, we have

(11) $$\frac{\partial^{2}\ln p\left(s^{\prime };s\right)}{\partial x^{2}}=-\frac{1}{2}\left[{\left(-\frac{\partial^{2}s}{\partial x^{2}}\right)}^{T}Q^{-1}\left(s^{\prime }-s\right)-{\left(s^{\prime }-s\right)}^{T}Q^{-1}\frac{\partial^{2}s}{\partial x^{2}}+2{\left(\frac{\partial s}{\partial x}\right)}^{T}Q^{-1}\frac{\partial s}{\partial x}\right],$$

(12) $$\frac{\partial^{2}\ln p\left(s^{\prime };s\right)}{\partial x\partial y}=-\frac{1}{2}\left[{\left(-\frac{\partial^{2}s}{\partial x\partial y}\right)}^{T}Q^{-1}\left(s^{\prime }-s\right)-{\left(s^{\prime }-s\right)}^{T}Q^{-1}\frac{\partial^{2}s}{\partial x\partial y}+2{\left(\frac{\partial s}{\partial x}\right)}^{T}Q^{-1}\frac{\partial s}{\partial y}\right],$$

(13) $$\frac{\partial^{2}\ln p\left(s^{\prime };s\right)}{\partial y^{2}}=-\frac{1}{2}\left[{\left(-\frac{\partial^{2}s}{\partial y^{2}}\right)}^{T}Q^{-1}\left(s^{\prime }-s\right)-{\left(s^{\prime }-s\right)}^{T}Q^{-1}\frac{\partial^{2}s}{\partial y^{2}}+2{\left(\frac{\partial s}{\partial y}\right)}^{T}Q^{-1}\frac{\partial s}{\partial y}\right].$$

The expectations of Equations (11)–(13) are as follows:

(14) $$E\left[\frac{\partial^{2}\ln p\left(s^{\prime };s\right)}{\partial x^{2}}\right]=-\left[{\left(\frac{\partial s}{\partial x}\right)}^{T}Q^{-1}\frac{\partial s}{\partial x}\right],$$

(15) $$E\left[\frac{\partial^{2}\ln p\left(s^{\prime };s\right)}{\partial x\partial y}\right]=-\left[{\left(\frac{\partial s}{\partial x}\right)}^{T}Q^{-1}\frac{\partial s}{\partial y}\right],$$

(16) $$E\left[\frac{\partial^{2}\ln p\left(s^{\prime };s\right)}{\partial y^{2}}\right]=-\left[{\left(\frac{\partial s}{\partial y}\right)}^{T}Q^{-1}\frac{\partial s}{\partial y}\right].$$

Then, the Fisher information matrix FIM(s) for the localization parameters can be denoted as

(17) $$\mathrm{FIM}\left(s\right)=\left[\begin{array}{cc} E\left[\frac{\partial^{2}\ln p\left(s^{\prime };s\right)}{\partial x^{2}}\right] & E\left[\frac{\partial^{2}\ln p\left(s^{\prime };s\right)}{\partial x\partial y}\right]\\ E\left[\frac{\partial^{2}\ln p\left(s^{\prime };s\right)}{\partial x\partial y}\right] & E\left[\frac{\partial^{2}\ln p\left(s^{\prime };s\right)}{\partial y^{2}}\right] \end{array}\right]=\left[\begin{array}{cc} {\left(\frac{\partial s}{\partial x}\right)}^{T}Q^{-1}\frac{\partial s}{\partial x} & {\left(\frac{\partial s}{\partial x}\right)}^{T}Q^{-1}\frac{\partial s}{\partial y}\\ {\left(\frac{\partial s}{\partial x}\right)}^{T}Q^{-1}\frac{\partial s}{\partial y} & {\left(\frac{\partial s}{\partial y}\right)}^{T}Q^{-1}\frac{\partial s}{\partial y} \end{array}\right].$$

Thus, the CRLB matrix for target position estimations could be obtained by taking the inverse of the Fisher information matrix FIM(s)

(18) $$\mathrm{CRLB}\left(s\right)={\mathrm{FIM}}^{-1}\left(s\right).$$

### 2.3. Optimization Problem Formation

In this paper, we formulate two optimization problems for multi-target passive localization via UAVs: the node selection problem and the path optimization problem.

For a swarm of *n* UAVs and *m* targets, the node selection problem aims to optimally divide the *n* UAVs into *m* groups to minimize the average trace of the derived CRLB for each group’s UAVs localization results at this moment:

(19) $$\begin{array}{cc} \; & \min_{G_{1},\ldots ,G_{m}}\frac{1}{m}\sum_{j=1}^{m},\mathrm{Tr}{\left({\mathrm{CRLB}}_{t}\right)}_{j} \end{array},$$

where $G_{j}$ represents the coordinate matrix of all UAVs assigned to target *j*, and $\mathrm{Tr}{\left({\mathrm{CRLB}}_{t}\right)}_{j}$ is the trace of the CRLB matrix of the localization for the *j*th target at the current time step.

For each group of UAVs, the path optimization problem aims to determine the optimal position for each UAV at the next time step to minimize the trace of the derived CRLB, while satisfying the dynamic constraints for the UAV constraints:

(20) $$\begin{array}{cc} \; & \min_{G_{j}^{t+1}}\arg\mathrm{Tr}{\left({\mathrm{CRLB}}_{t+1}\right)}_{j} \end{array},$$

where $G_{i,j}^{t+1}$ represents the coordinate matrix of all UAVs assigned to target *j* at the next time step, and $\mathrm{Tr}{\left({\mathrm{CRLB}}_{t+1}\right)}_{j}$ is the prediction trace of the CRLB matrix of the localization result for the target *j* at the next time step.

In the following sections, we will introduce the detailed process of the proposed CRLB-based node selection method and the proposed CRLB-based path optimization method. Then, to accelerate the searching process for the optimal UAV positions, the PSO algorithm is introduced and applied to solve the path optimization problem.

## 3. CRLB-Based Node Selection Method

In this section, a CRLB-based node selection method is proposed to optimally divide UAVs into groups for localizing different targets, improving localization accuracies. Firstly, we describe the node selection problem and give the constraints of the problem. Then, the optimization model is formulated based on the derived CRLB, and the exhaustive method is deployed to minimize the average CRLB trace and obtain the optimal grouping scheme.

### 3.1. Problem Description

Consider a surveillance scenario with *m* stationary targets in a two-dimensional plane. *n* UAVs equipped with passive radars need to be divided properly into several groups for multi-target localization, and the number of UAVs of each groups for the *j*th target is $\lambda_{j}\left(j=1,2,\ldots ,m\right)$. The random assignment of UAVs may lead to suboptimal resource utilization and degraded localization accuracy. Therefore, we develop a systematic method for UAV grouping that ensures optimal performance.

### 3.2. Constraint Analysis

Since the Chan-TDOA method needs at least 3 UAVs to locate a target in a two-dimensional plane. Thus, the number of UAVs of each group should be more than 3. That is,

(21) $$\lambda_{j}\geq 3.$$

### 3.3. Node Selection Optimization Model

The objective of the node selection problem is to minimize the average CRLB trace across all targets, and the mathematical model can be denoted as follows

(22) $$\begin{array}{cc} \; & \min_{G_{1},\ldots ,G_{m}}\sum_{j=1}^{m}\mathrm{Tr}{\left({\mathrm{CRLB}}_{t}\right)}_{j}\\ \; & \mathrm{subject}\;\mathrm{to}\;\lambda_{j}\geq 3 \end{array},$$

where $G_{j}\left(j=1,2,\ldots ,m\right)$ represents the set of UAVs assigned to target *j*, and $\mathrm{Tr}{\left({\mathrm{CRLB}}_{t}\right)}_{j}$ denotes the trace of the CRLB matrix for target *j* at the current time step.

### 3.4. Node Selection Algorithm Design

The CRLB-based node selection algorithm provides a systematic approach for optimally grouping UAVs to locate different targets while minimizing localization errors. The algorithm employs a two-stage exhaustive search method: Firstly, it enumerates all possible combinations to partition *n* UAVs into *m* groups, where each group must contain at least 3 UAVs ($\lambda_{j}\geq 3$) to ensure effective target localization. For each configuration in this solution space, the algorithm computes the CRLB matrices for all targets and calculates their average trace as a metric of localization accuracy, selecting the one that minimizes this metric as the globally optimal solution. The detailed implementation of the node selection method is presented in Algorithm 1.
**Algorithm 1:** CRLB-based node selection algorithm
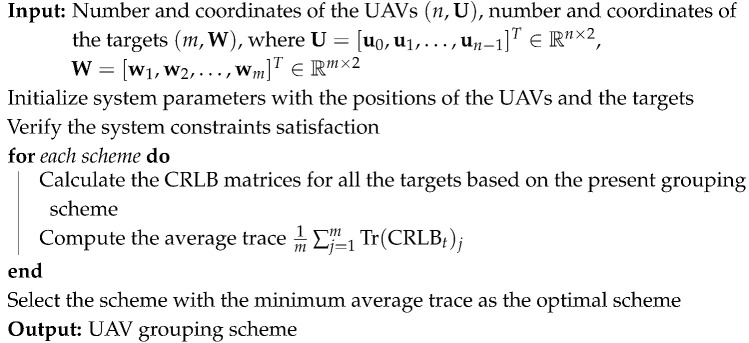


## 4. CRLB-Based Path Optimization Method

In this section, we presents a CRLB-based path optimization method to dynamically adjust the UAVs to the optimal positions, enhancing the target localization accuracy. For the sake of a clear presentation in this section, we consider path optimization for a group of UAVs localizing a certain target after node selection; thus, the UAVs have been optimally grouped for each target. In this case, for the convenience of description, target index *j* is omitted in the following derivations.

### 4.1. Problem Description

Consider a group of λ UAVs localizing a target at the current time *t*. The path planning updates are governed by the optimization time interval Δt. For the convenience of description, it is assumed that each UAV maintains a constant flight speed *v* during the path planning process. The variables that need to be optimized are the deflection angles $\phi_{i}^{t}\;\left(i=1,2,\ldots ,\lambda \right)$ of the UAVs, which represent the angles between the heading direction of the UAVs and the positive x-axis (measured counterclockwise), and once the deflection angles are optimized, the positions of the UAVs at time t+1 are determined. Thus, optimizing the deflection angles $\phi_{i}^{t}\;\left(i=1,2,\ldots ,\lambda \right)$ of the UAVs equals optimizing the positions of the UAVs. We assume that the positions of the λ UAVs at time *t* are G, which can be structured as

(23) $$G^{t}={\left[\begin{array}{c} {\left(u_{1}^{t}\right)}^{T}\\ {\left(u_{2}^{t}\right)}^{T}\\ \vdots \\ {\left(u_{i}^{t}\right)}^{T}\\ \vdots \\ {\left(u_{\lambda }^{t}\right)}^{T} \end{array}\right]}_{\lambda \times 2},$$

where $u_{i}^{t}={\left[x_{i}^{t},y_{i}^{t}\right]}^{T}$ denotes the two-dimensional coordinates of the *i*th UAV at time *t*, and λ represents the total number of the UAVs in the group.

### 4.2. Constraint Analysis

To ensure the physical feasibility of the UAVs’ movements, we consider four fundamental constraints:

(1) The turning radius constraint: Due to the mechanical limitations of UAVs, the actual turning radius $L_{i}^{t}$ must be greater than the minimum allowable turning radius $L_{\min}$ (which is determined by the system parameters of the UAV). The minimum allowable turning radius is as follows:

(24) $$L_{i}^{t}\geq L_{\min},$$

The turning radius $L_{i}^{t}$ can be determined by the UAV’s movement during the path planning time interval Δt. We assume that within the Δt time interval, the turning arc angle of the UAV is $\theta_{i}^{t}$; then, the turning arc can be approximated as a circular arc that is shown as follows:

(25) $$L_{i}^{t}\theta_{i}^{t}=v\Delta t,$$

**Figure 3:**
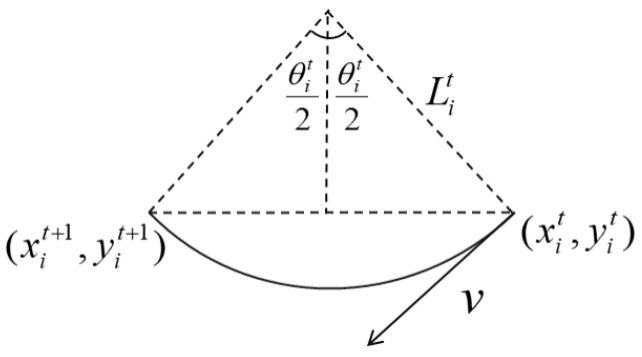
Diagram of UAV motion constraints. The UAV moves from position $\left(x_{i}^{t},y_{i}^{t}\right)$ to $\left(x_{i}^{t+1},y_{i}^{t+1}\right)$ along a circular arc, where $L_{i}^{t}$ represents the path length, $\theta_{i}^{t}$ is the turning angle, and *v* denotes the velocity vector of the UAV.

According to the geometric relationship shown in Figure 3, we obtain

(26) $$L_{i}^{t}\sin\left(\frac{\theta_{i}^{t}}{2}\right)=\frac{1}{2}∥u_{i}^{t+1}-u_{i}^{t}∥,$$

where $\sin\left(\theta_{i}^{t}/2\right)$ denotes the sine function. Combining (25) with (26), we can solve the turning arc angle $\theta_{i}^{t}$ and turning radius $L_{i}^{t}$.

(2) The minimum approach distance constraint: the distance $r_{i}^{t}$ between each UAV and the target must not be less than the minimum approach distance $d_{min}$, and the distance $r_{i}^{t}$ should satisfy

(27) $$r_{i}^{t}\geq d_{min},$$

where $r_{i}^{t}$ represents the distance between the *i*th UAV and the target at time *t*.

(3) The deflection angle constraint: To ensure that the UAVs head toward the target, the heading direction of the UAV *i* at time *t* must be within the left and right angle $\Delta \alpha_{i}^{t}$ range along the line connecting it to the target. Therefore, the deflection angle $\phi_{i}^{t}$ of the UAV *i* at time *t* must satisfy

(28) $$\phi_{i}^{t}\in \left(\alpha_{i}^{t}+\Delta \alpha_{i}^{t},\alpha_{i}^{t}-\Delta \alpha_{i}^{t}\right),$$

where the parameters are defined by

(29) $$\left\{\begin{array}{c} \alpha_{i}^{t}=\arctan\frac{y_{i}^{t}-y^{t}}{x_{i}^{t}-x^{t}}\\ \Delta \alpha_{i}^{t}=\pi \frac{r_{i}^{0}-r_{i}^{t}}{r_{i}^{0}} \end{array}\right.,$$

where $\alpha_{i}^{t}$ represents the azimuth angle of the target related to the *i*th UAV, and $\Delta \alpha_{i}^{t}$ is the adaptive adjustment range.

(4) No-fly-zone (NFZ) constraint: To ensure operational safety and comply with airspace restrictions, the UAVs should not fly in the NFZ

(30) $$\left(x_{i}^{t},y_{i}^{t}\right)\notin S_{\mathrm{NFZ}},$$

where $\left(x_{i}^{t},y_{i}^{t}\right)$ represents the position coordinates of the *i*th UAV at time *t*, and $S_{\mathrm{NFZ}}$ represents the complete set of restricted points, which is the union of all these individual NFZ and can be represented as

(31) $$S_{\mathrm{NFZ}}=\underset{k=1}{\overset{p}{\cup }}S_{k},$$

where *p* is the number of the individual NFZ.

### 4.3. Path Optimization Model

Based on the problem formulation and the constraints derived above, the mathematical model for UAV path optimization can be described as

(32) $$\begin{array}{cc} \; & \min_{G^{t+1}}\arg\mathrm{Tr}\left({\mathrm{CRLB}}_{t+1}\right)\\ \; & \mathrm{subject}\;\mathrm{to}\left\{\begin{array}{c} L_{i}^{t}\geq L_{\min}\\ r_{i}^{t}\geq d_{\min}\\ \phi_{i}^{t}\in \left(\alpha_{i}^{t}+\Delta \alpha_{i}^{t},\alpha_{i}^{t}-\Delta \alpha_{i}^{t}\right)\\ \left(x_{i}^{t},y_{i}^{t}\right)\notin S_{\mathrm{NFZ}} \end{array}\right. \end{array},$$

where $G_{i}^{t+1}$ represents the planed position of the UAVs that are assigned to the target at the next time step, and $\mathrm{Tr}({\mathrm{CRLB}}_{t+1})$ is the trace of the predicted CRLB matrix for the localization of the target at the next time step.

### 4.4. Path Optimization Algorithm Design

To quickly search the planned optimal positions for the UAVs to localize the target at the next time step, in this paper, the PSO algorithm is deployed.

Suppose there are *S* particles searching in each iteration, and each particle *s* (1≤s≤S) has information about its own best position $p^{s}$, which it has never visited; its current position $\eta^{s}$; its velocity $u^{s}$; and the global best solution $p^{g}$ found by all particles. Specifically, in the γth dimension, the movement of each particle is influenced by its own best position $p_{\gamma }^{s}$, the global best position $p_{\gamma }^{g}$, and its current velocity $u_{\gamma }^{s}$. The velocity and position are updated as follows, where *k* denotes the iteration step for the particle.

(33) $$u^{s}\left(k+1\right)=\omega \left(k\right)\times u^{s}\left(k\right)+c_{1}\left(k\right)l_{1}\left(k\right)\left(p^{s}\left(k\right)-\eta^{s}\left(k\right)\right)+c_{2}\left(k\right)l_{2}\left(k\right)\left(p^{g}\left(k\right)-\eta^{s}\left(k\right)\right),$$

(34) $$\eta^{s}\left(k+1\right)=\eta^{s}\left(k\right)+u^{s}\left(k+1\right),$$

where ω(k) is the inertia weight, which decreases with iteration time as $\omega \left(k\right)=0.9-0.5\left(k/T_{max}\right)$. $T_{max}$ is the maximum number of iterations. $c_{1}\left(k\right)$ and $c_{2}\left(k\right)$ are individual learning and global learning factors, which vary with iteration time as $c_{1}\left(k\right)=2.5-2\left(k/T_{max}\right)$ and $c_{2}\left(k\right)=0.5+2\left(k/T_{max}\right)$ respectively. $l_{1}\left(k\right)$ and $l_{2}\left(k\right)$ are random independent variables in the range [0,1]. The velocity of particle in each dimension is limited by a fixed $V_{max}$. The current position of each particle represents its current proposed solution. The performance of each solution is evaluated via a fitness function (denoted by F). In this algorithm, each particle is a 2×λ-dimensional vector that contains all positions of the λ UAVs in two-dimension space. For instance, the expression of the *s*th particle is

(35) $$\eta^{s}=\Phi^{s}=\left[\phi_{1}^{s},\phi_{2}^{s},\ldots ,\phi_{\lambda }^{s}\right],$$

where $\phi_{i}^{s}\left(i=1,2,\ldots ,\lambda \right)$ denotes the deflection angle of the *i*th UAV in the *s*th particle, and λ is the number of UAVs. For initialization, we randomly place all UAVs in a certain region. The velocity and position of every particle will be updated in each iteration. The fitness function of the *s*th particle is as follows:

(36) $$f^{s}\left(k\right)=F\left(\eta^{s}\left(k\right)\right)=\mathrm{Tr}\left[\mathrm{CRLB}\left(\Phi^{s}\right)\right],$$

where $\eta^{s}\left(k\right)$ denotes the current position of the *s*th particle, and $p^{g}\left(k\right)$ can be updated as

(37) $$p^{s}\left(k+1\right)=\left\{\begin{array}{cc} \eta^{s}\left(k+1\right), & \mathrm{if}\;f^{s}\left(k+1\right)<F\left(p^{s}\left(k\right)\right)\\ p^{s}\left(k\right), & \mathrm{if}\;f^{s}\left(k+1\right)\geq F\left(p^{s}\left(k\right)\right) \end{array}\right.,$$

(38) $$p^{g}\left(k+1\right)=\arg\;\max_{1\leq s\leq S}F\left(p^{s}\left(k+1\right)\right).$$

When $k=T_{max}$, the iteration will stop and output the optimization result; thus, the optimal UAV positions will be obtained. Here, a summary process of the path optimization is given in Algorithm 2.
**Algorithm 2:** CRLB-based path optimization via PSO
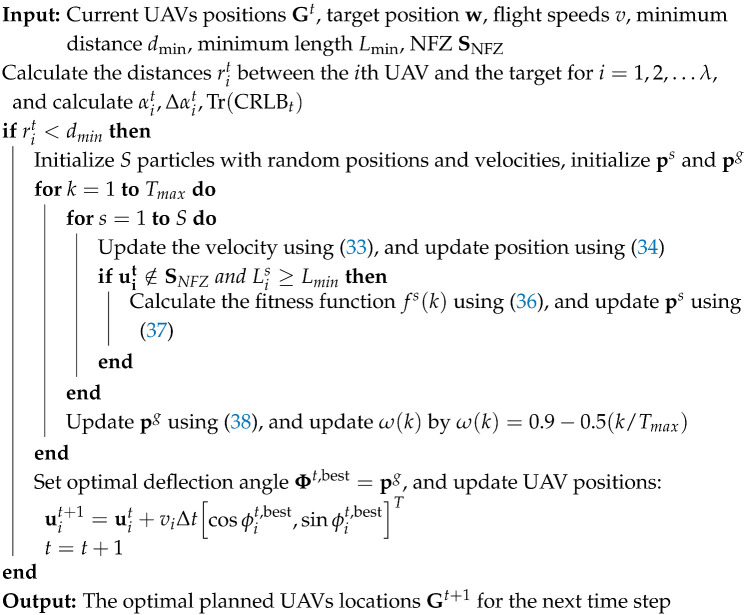


## 5. Numerical Results

In this section, we present numerical simulations to verify the validity and effectiveness of the proposals in this paper. First, we set the parameters and the initial topology structure of the UAVs and the targets. Then, the simulation results of the node selection method and the path optimization method are given. Finally, the time costs of the PSO algorithm and the exhaustive algorithm are compared to verify the effectiveness of the PSO algorithm.

### 5.1. Simulation Parameters and the Topology Structure Set

**Figure 4:**
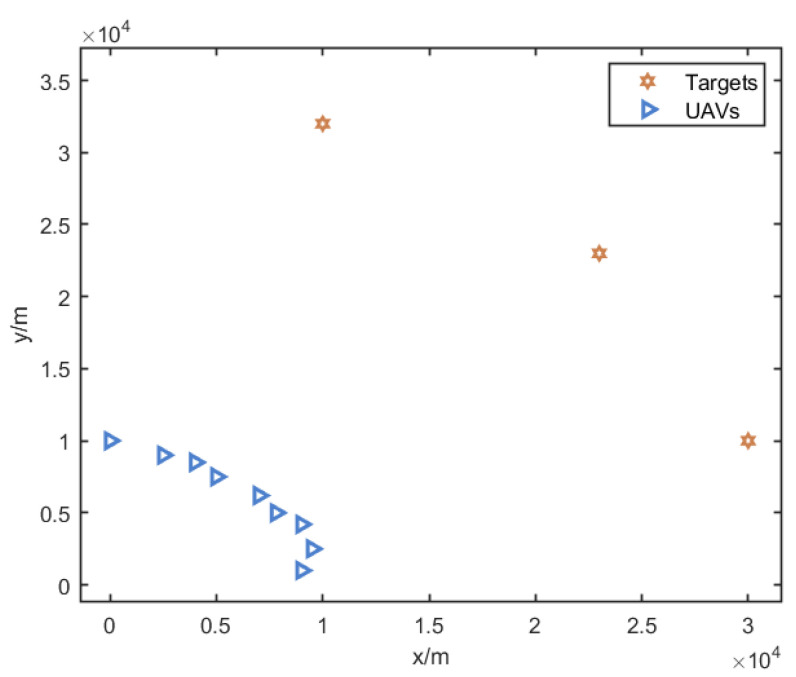
Initial positions of the UAVs and the targets. The blue triangles represent the initial positions of the 9 UAVs (UAV0–UAV8), and the orange stars represent the initial positions of the 3 targets.

Consider that there are nine UAVs (n=9) and three targets (m=3) in a two-dimensional plane. The positions of the UAVs are [0, 10,000] m, [2500, 9000] m, [4000, 8500] m, [5000, 7500] m, [7000, 6200] m, [7800, 5000] m, [9000, 4200] m, [9500, 2500] m, and [9000, 1000] m. The positions of the targets are [10,000, 32,000] m, [23,000, 23,000] m, and [30,000, 10,000] m. The UAVs’ minimum turning radius is set to be $r_{\min}=100$ m, and the minimum approach distance is set to be $d_{\min}=5000$ m. The initial topology of the UAVs and the targets is shown in Figure 4.

### 5.2. Simulation Results of the Node Selection Algorithm

**Figure 5:**
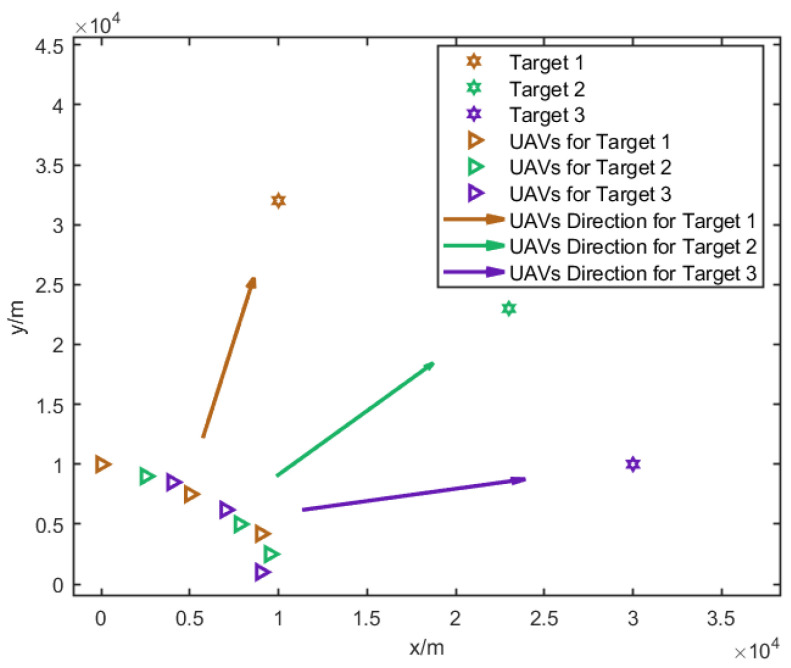
UAV grouping results and their movement directions after node selection. Different colors represent different UAV groups assigned to their respective targets, and arrows indicate the planned movement directions.

In this paper, it is assumed that at each time step, three UAVs are assigned to a target; thus, the UAVs will be divided into three groups to localize for three targets. The result of the CRLB-based node selection is shown in Figure 5.

To evaluate the performance of the node selection, the root mean square error (RMSE) is used as a localization accuracy metric, and the performance comparisons between the proposed CRLB-based node selection algorithm with the random selection method are made. The RMSE can be obtained by

(39) $$\mathrm{RMSE}=\sqrt{\frac{1}{K}\sum_{q=1}^{K}\left[{\left(x_{q}^{\prime }-x\right)}^{2}+{\left(y_{q}^{\prime }-y\right)}^{2}\right]}.$$

A total of 1000 independent Monte Carlo experiments (K=1000) were conducted to complete the comparisons. For the convenience of providing descriptions, in the following part, the proposed CRLB-based node selection algorithm is called the proposed algorithm for short, and the random node selection method is called the traditional algorithm for short.

From Table 1, it can be seen that the RMSEs of the proposed algorithm are much lower than that of the traditional algorithm. The RMSEs of the three different target based on the proposed algorithm decrease about 20–30% compared to the traditional algorithm. The average execution time of the proposed algorithm is 50.62 ms per iteration. The results illustrate that by efficient node selection, the target localization accuracy can be improved at a large scale.

### 5.3. Simulation Results of the CRLB-Based Path Optimization Method

Through the node selection method, the UAVs have been divided into several groups for different target localizations. In this part, for each UAV group, we analyze the performance of the CRLB-based path optimization algorithm. The PSO algorithm is implemented with 50 particles and 500 maximum iterations in a three-dimensional continuous solution space, where each dimension represents the deflection angle of each UAV. The cognitive factor $c_{1}$ decreases linearly from 2.5 to 0.5, and the social factor $c_{2}$ increases from 0.5 to 2.5, with an inertia weight *w* decreasing from 0.9 to 0.4. A total of 800 independent Monte Carlo experiments are conducted for the comparison between the proposed CRLB-based path optimization method and the fixed formation method. For the convenience of description, we call the proposed CRLB-based path optimization algorithm as the proposed algorithm for short and call the fixed formation algorithm the traditional algorithm for short.

**Figure 6:**
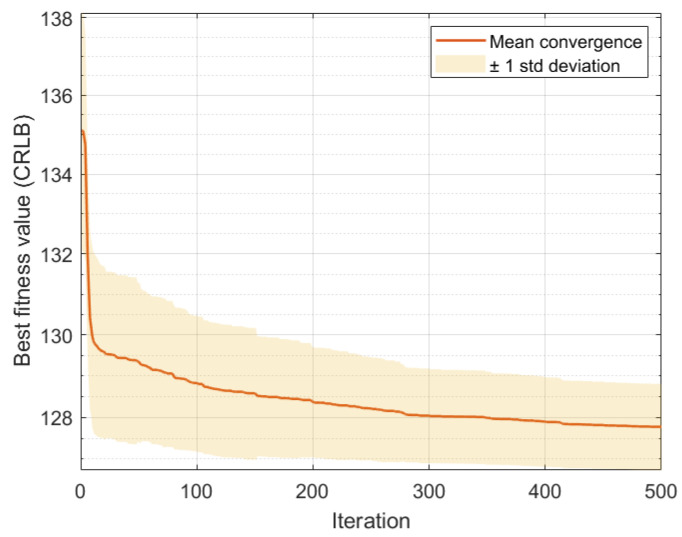
Evolution of best fitness value (CRLB) during PSO iterations for the first UAV group at the initial time step.

Figure 6 shows the average convergence behavior of the PSO algorithm over 50 independent runs during path optimization for the first UAV group targeting target 1 at the initial time step. The solid line represents the mean convergence curve, while the shaded area indicates one standard deviation above and below the mean value. The algorithm reliably achieves stable convergence after approximately 300 iterations, with the final average CRLB value reaching 127.8 $m^{2}$ with a standard deviation of approximately ±1.2 $m^{2}$. This stable convergence pattern across multiple runs validates the robustness of the proposed PSO parameter settings in consistently finding high-quality solutions.

**Figure 7:**
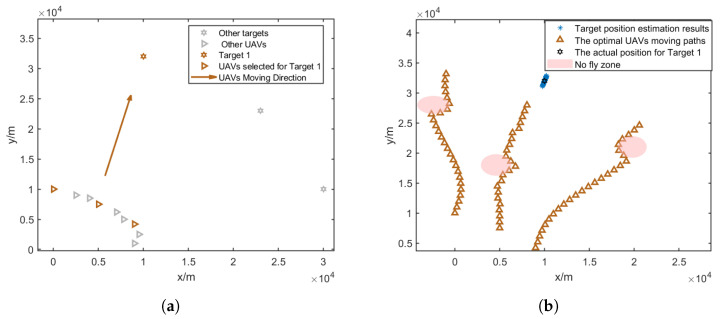
Three selected UAVs localizing target 1 and their moving direction. (**a**) Movement direction of the three selected UAV groups for target 1. (**b**) Trajectories of the three selected UAV groups for target 1.

**Figure 8:**
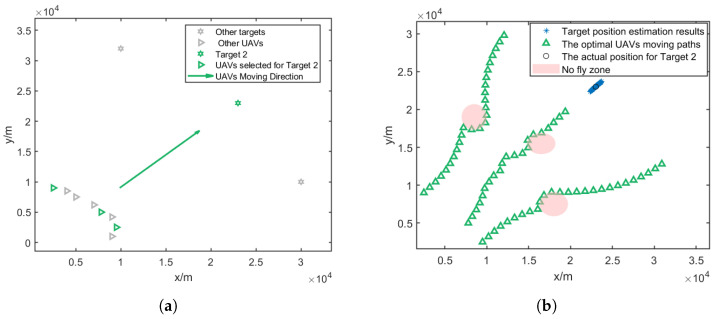
Three selected UAVs localizing target 2 and their moving directions. (**a**) Movement direction of the three selected UAV groups for target 2. (**b**) Trajectories of the three selected UAV groups for target 2.

**Figure 9:**
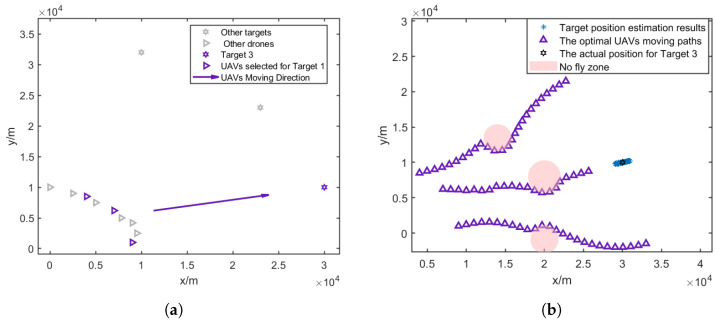
Three selected UAVs localizing target 3 and their moving direction. (**a**) Movement direction of the three selected UAV groups for target 3. (**b**) Trajectories of the three selected UAV groups for target 3.

**Figure 10:**
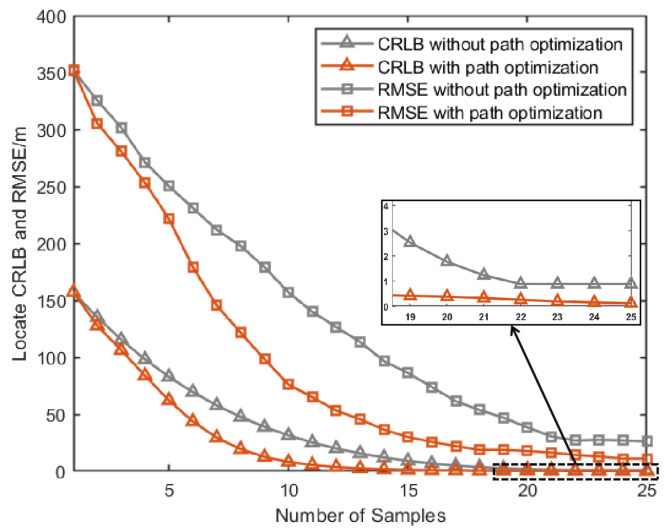
Comparison of CRLB and RMSE under path optimization versus fixed configuration for target 1.

**Figure 11:**
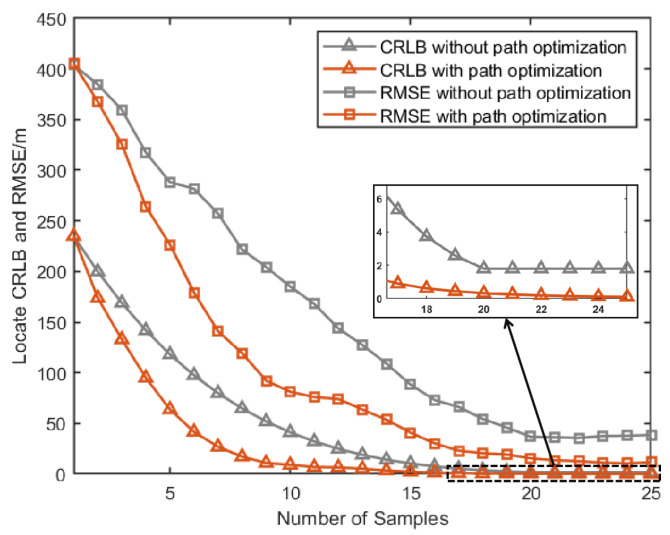
Comparison of RMSE and CRLB between the proposed path optimization method and the traditional method.

**Figure 12:**
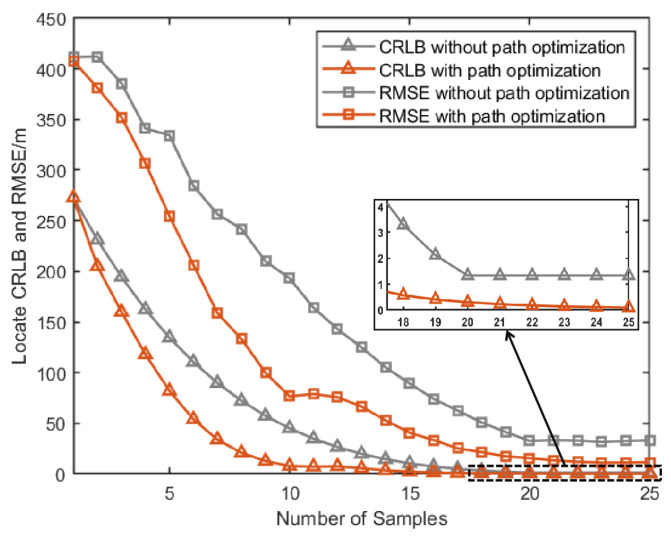
Comparison of RMSE and CRLB between the proposed path optimization method and the traditional method.

Figure 7a, Figure 8a and Figure 9a give the topology of the UAVs and the target for each UAV group. Figure 7b, Figure 8b and Figure 9b show the UAVs’ motion paths based on the proposed path optimization method, and the RMSE and CRLB of the localization for each target are shown in Figure 10, Figure 11 and Figure 12.

From Figure 10, Figure 11 and Figure 12, it can be seen that for all three groups of UAVs, the RMSE and CRLB of the proposed path optimization method is lower than that of the traditional method. In Figure 10, the average RMSE for the proposed method is lower by about 43.37% than the traditional method, and the average CRLB for the proposed method is lower by about 62.99% than the traditional method. In Figure 11, the average RMSE for the proposed method is lower by about 47.59% than the traditional method, and the average CRLB for the proposed method is lower by about 68.65% than the traditional method. And in Figure 12, the average RMSE for the proposed method is lower by about 45.01% than the traditional method, and the average CRLB for the proposed method is lower by about 66.68% than the traditional method. Moreover, the proposed method cab avoid the NFZ adaptively, while the fixed formation method in this paper is assumed to be unable to avoid the NFZ.

To verify the effectiveness of the PSO algorithm used in this paper, we compared PSO with three other optimization algorithms: GA, ACO, and the traditional exhaustive method. These algorithms are used to search for Φ (the optimal UAV positions) at each time step to obtain the minimum trace of CRLB for the next time step. The parameter settings for the algorithms are shown in Table 2. GA operates in continuous space with 20 individuals, employing a crossover rate of 0.8 and a mutation rate of 0.1. The PSO parameter settings are the same as in the previous experiment, while ACO discretizes the search space into 20 points per dimension, using 50 ants with pheromone-based search strategies. All algorithms are set to a maximum of 500 iterations with different early stopping criteria and optimization strategies to enhance search efficiency. For the exhaustive method, the angle range is discretized into 50 equally spaced points, resulting in $50^{3}$ combinations that need to be evaluated at each time step to find the optimal waypoints resulting the minimum trace of CRLB.

**Figure 13:**
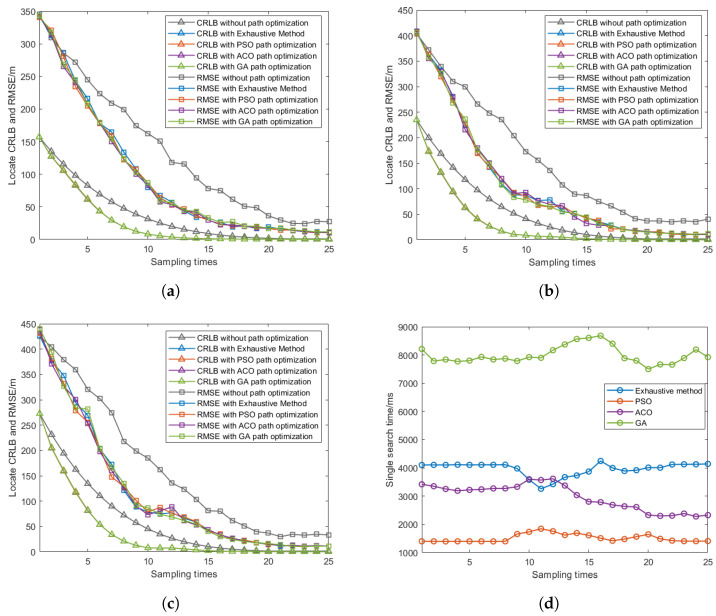
CRLB comparison and single-iteration computation time comparisons with different optimization algorithms localizing different targets. (**a**) CRLB comparison with different optimization algorithms localizing target 1. (**b**) CRLB comparison with different optimization algorithms localizing target 2. (**c**) CRLB comparison with different optimization algorithms localizing target 3. (**d**) Single-iteration computation time comparisons between different optimization methods.

As shown in Figure 13a–c, we present the CRLB and RMSE performance comparisons for five different methods: the fixed formation method (without path optimization) and four optimization methods (Exhaustive, PSO, ACO, and GA) localizing three different targets. The simulation results demonstrate that for localizing different targets, the four optimization approaches present similar convergence patterns in both CRLB and RMSE metrics, with their curves nearly overlapping throughout the localization process, and they all achieve lower CRLB and RMSE values compared to the fixed formation method. Figure 13d shows the computational time comparisons for a single iteration between different optimization methods. The results reveal that to realize the approximate localizing performance, the PSO algorithm maintains the lowest computation time consistently across all sampling times, while GA shows the highest computational burden and ACO demonstrates moderate computational requirements, positioned between PSO and the exhaustive method.

Table 3 shows the average percentage reduction in the CRLB and RMSE of path optimization algorithms (Exhaustive, PSO, ACO, and GA) compared to the fixed formation method. Specifically, the CRLB reduction for Exhaustive, PSO, ACO, and GA is 66.70%, 66.66%, 66.77%, and 66.57%, respectively, compared to the fixed formation method, while the RMSE reduction is 44.24%, 44.99%, 45.02%, and 44.64%, respectively, compared to the fixed formation method, indicating that with path optimization methods, the localization performance of the UAVs can be improved at a large scale. Table 3 gives the comparisons of the average single-iteration computation time between the four path optimization methods, with PSO taking 1.34 s, ACO taking 3.05 s, the Exhaustive method taking 4.28 s, and GA taking 7.76 s. It is evident that the PSO method applied in this paper takes the shortest computation time, followed by ACO and then the exhaustive method, while GA requires significantly more time. These results indicate that while all methods can achieve similar localization performance, and PSO offers the best computational efficiency, making it particularly suitable for real-time UAV path planning applications.

As shown in Table 3, while all algorithms achieve similar CRLB and RMSE reductions compared to the fixed formation method, PSO demonstrates superior computational efficiency. These results suggest that PSO provides the best balance between computational efficiency and optimization performance, making it particularly suitable for real-time UAV formation, quickly responding to environmental changes.

The performance of the proposed algorithm in terms of localization accuracy and computational efficiency can be explained from two aspects. Firstly, while the traditional fixed formation method adopts predetermined UAV configurations without considering the impact of flight paths on localization performance, the CRLB-based path optimization algorithm continuously adjusts UAV flight paths to minimize the trace of CRLB. This optimization mechanism enables UAVs to select better observation angles, thereby obtaining more accurate measurements, reducing measurement errors, and improving localization accuracy, as evidenced by the lower RMSE values shown in Figure 13a–c. Secondly, among the four optimization algorithms, PSO converges to optimal solutions in less time with higher computational efficiency. This is because GA, ACO, and Exhaustive methods require global information and involve complex computations, while PSO’s characteristic of making decisions based on local relative measurements better meet real-time control requirements, resulting in shorter computation times, as shown in Figure 13d.

### 5.4. Impact Analysis of Minimum Turning Radius

To thoroughly investigate the impact of the minimum turning radius on path optimization and localization accuracy, we conducted comparative experiments with different minimum turning radii while keeping other parameters constant. Three UAVs were initially deployed at coordinates [0, 10,000] m, [5000, 7500] m, and [9000, 4200] m, with the target located at [50,000, 32,000] m. Using the PSO optimization algorithm introduced in Section 4, we set the minimum turning radius $L_{min}$ to 5000 m, 7500 m, and 10,000 m to examine its influence on UAV path optimization and localization performance. In total, 800 Monte Carlo simulations were performed to ensure the statistical significance of the results.

**Figure 14:**
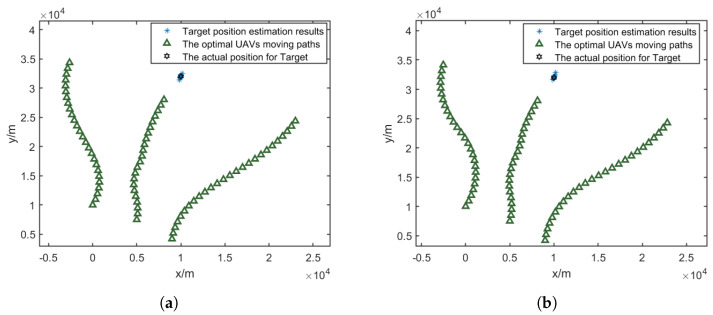

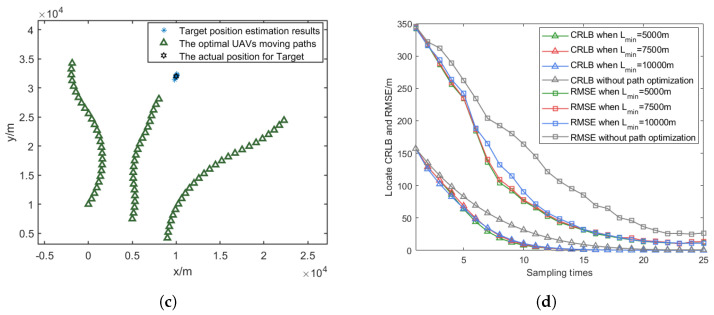
Optimized UAV paths under different minimum turning radii and their RMSE comparisons. (**a**) $L_{min}$ = 5000 m. (**b**) $L_{min}$ = 7500 m. (**c**) $L_{min}$ = 10,000 m. (**d**) Comparison of RMSE and CRLB.

As shown in Figure 14a–c, the optimized UAV paths exhibit distinct characteristics under different minimum turning radius constraints. With increasing $L_{min}$, the UAVs require wider turning arcs to change their flight directions, resulting in longer path lengths and more gradual course adjustments.

Figure 14d and Table 4 demonstrate the impact of turning radius constraints on localization accuracy. The simulation results show that a smaller turning radius allows for more flexible path planning and better localization performance. When $L_{min}$ increases from 5000 m to 10,000 m, the CRLB reduction deceases by 2.86 percentage points (from 64.57% CRLB reduction compared to the fixed formation method to 61.71%), while the RMSE reduction deceases by 3.59 percentage points (from 45.38% RMSE reduction compared to the fixed formation method to 41.79%). This trend indicates that larger minimum turning radius constraints moderately impact the algorithm’s ability to optimize UAV paths for target localization, as they limit the UAVs’ maneuverability in adjusting their positions for optimal measurement geometry.

### 5.5. Impact Analysis of No-Fly-Zone Size

To evaluate the impact of no-fly-zone size on localization performance, we conducted comparative experiments with different no-fly-zone radii while maintaining other parameters constant. Three UAVs were initially deployed at coordinates [0, 10,000] m, [5000, 7500] m, and [9000, 4200] m, with the target located at [50,000, 32,000] m. Three circular no-fly zones were centered at [−2400, 28,000] m, [4600, 18,000] m, and [19,800, 21,000] m. Using the PSO optimization algorithm introduced in Section 4, we set the radii of no-fly zone $R_{NFZ}$ to 1000 m, 2000 m, and 3000 m to examine their influence on localization performance. Also, 800 Monte Carlo simulations were performed to ensure the statistical significance of the results.

**Figure 15:**
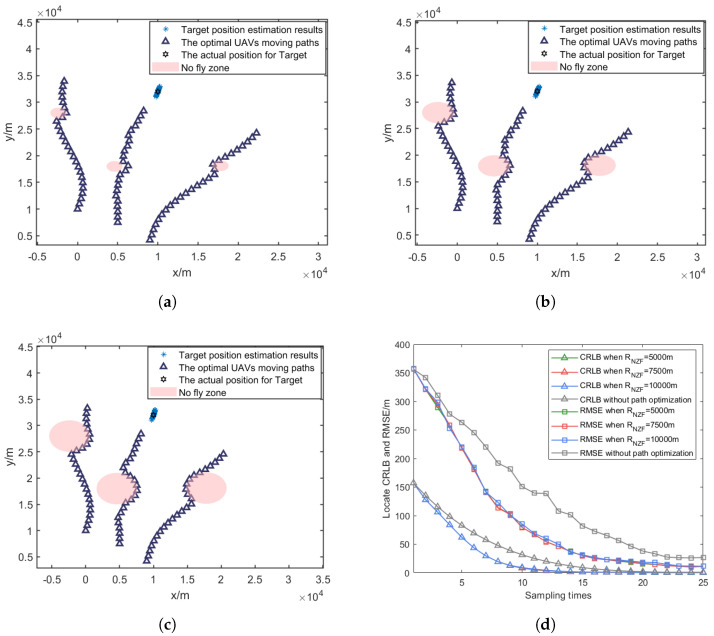
Optimized UAV paths and comparison of RMSE and CRLB under different no-fly-zone radii. (**a**) $R_{NFZ}$ = 1000 m. (**b**) $R_{NFZ}$ = 2000 m. (**c**) $R_{NFZ}$ = 3000 m. (**d**) Comparisons of RMSE and CRLB.

As shown in Figure 15a–c, the optimized UAV paths demonstrate different characteristics as $R_{NFZ}$ increases. Larger no-fly zones force the UAVs to take more substantial detours, resulting in longer path lengths and more constrained movement patterns.

Figure 15d and Table 5 illustrate the impact of no-fly-zone size on localization accuracy. The simulation results indicate that increasing $R_{NFZ}$ leads to degraded localization performance, as it restricts the available space for UAV path optimization solutions. When $R_{NFZ}$ increases from 1000 m to 3000 m, the CRLB reduction decreases by 3.94 percentage points (from 63.88% CRLB reduction compared to the fixed formation method to 59.94%), while the RMSE reduction decreases by 2.56 percentage points (from 45.02% RMSE reduction compared to the fixed formation method to 42.46%). This degradation in performance can be attributed to the reduced flexibility in configuring UAVs relative to the optimal measurement geometry, as larger restricted areas limit the available space for tactical maneuvers.

## 6. Conclusions

In this paper, we investigate the problem of node selection and path optimization for multi-target passive localization via UAVs. First, the Chan-TDOA target passive localization model is established.Then, the CRLB-based node selection algorithm is proposed to properly divide the UAVs into several groups localizing different targets, and the CRLB-based path optimization algorithm is proposed to search for the optimal UAV position configuration at each time step. The proposed path optimization method considers NFZ avoidance, ensuring both operational safety and localization accuracy. To improve the efficiency of path optimization, the PSO algorithm is applied to accelerate the searching process. Finally, numerical simulations were performed to verify the validity and effectiveness of the proposals in this paper.

## Figures and Tables

**Table 1:** RMSE comparisons between the proposed node selection method and the traditional method.

RMSE (Traditional) (m)	RMSE (Proposed) (m)	Percentage Reduction (%)
564.7680	352.0307	37.6681
532.7729	408.3508	23.3537
590.6810	427.4778	27.6297

**Table 2:** Parameter settings of the three optimization algorithms.

Parameter	PSO	GA	ACO
Size	50 particles	20 individuals	50 ants
Max iterations	500	500	500
Early stopping	Fitness < $1e^{-6}$	No improvement in	fitness < $1e^{-6}$
criterion		20 generations	
Special	$c_{1}$: 2.5 → 0.5	Crossover rate: 0.8	Evaporation rate: 0.1
parameters	$c_{2}$: 0.5 → 2.5	Mutation rate: 0.1	Influence factor (α): 1
	*w*: 0.9 → 0.4	Elite count: 2	Heuristic factor (β): 2
	Constriction: 0.729		Deposit factor (Q): 1
Optimization	Local search per	Elite retention	Local search per
strategy	10 iterations;		10 iterations;
	Reinitialize 10%		Space discretization:
	Particles per 20 iter.		50 points

**Table 3:** Performance comparison between different optimization methods.

Metric	Exhaustive	PSO	ACO	GA
CRLB reduction (%)	66.70	66.66	66.77	66.57
RMSE reduction (%)	44.24	44.99	45.02	44.64
Single iteration computation time(s)	4.28	1.34	3.05	7.76

**Table 4:** Average performance improvement compared to the traditional method.

Minimum Turning Radius	CRLB Reduction	RMSE Reduction
$\left(L_{\min}\right)$	(%)	(%)
5000 m	64.57	45.38
7500 m	62.15	43.14
10,000 m	61.71	41.79

**Table 5:** Average performance under different no-fly-zone radii.

No-Fly-Zone Radius	CRLB Reduction	RMSE Reduction
$\left(R_{\mathrm{NFZ}}\right)$	(%)	(%)
1000 m	63.88	45.02
2000 m	62.51	44.93
3000 m	59.94	42.46

## Data Availability

Due to privacy constraints, we are unable to share the data utilized in this study for submission purposes. We assure you that the research methodology and results outlined in this paper comply with ethical guidelines and have been executed in line with recognized research protocols.

## Appendix A. Mathematical Derivations of Chan-TDOA Algorithm

Since the UAVs (n≥3) are localizing the same target, we omit the superscript j in this part for convenience. Therefore, the target’s location is denoted as ${\left[x,y\right]}^{T}$. Then, the Chan-TDOA algorithm can be presented as follows:

For the convenience of comprehension, we first give the definitions for the important variables that would be used in subsequent derivations:

(1) $M_{i}=x_{i}^{2}+y_{i}^{2}$ represents the square of the location of the *i*th UAV (including i=0).

(2) $x_{i,0}=x_{i}-x_{0}$ and $y_{i,0}=y_{i}-y_{0}$ denote the relative locations of the *i*th UAV with respect to the master base station.

(3) $r_{i,0}=r_{i}-r_{0}$ indicates the difference in distance from the *i*th UAV to the target and from the master base station to the target.

(4) $\tau_{i,0}=\tau_{i}-\tau_{0}$ represents the difference in the times at which the signal was received by the *i*th UAV and received by the master base station.

(5) $\sigma_{i,0}^{2}=\sigma_{i}^{2}+\sigma_{0}^{2}$ represents the variance in the TDOA measurement for the target.

It is apparent that

(A1) $$r_{i}=r_{i,0}+r_{0}.$$

Squaring both sides of (A1), we obtain the following:

(A2) $$r_{i}^{2}=r_{i,0}^{2}+r_{0}^{2}+2r_{i,0}r_{0},$$

Substituting (1) into (A2) yields (A3):

(A3) $$x_{i,0}x+y_{i,0}y+r_{i,0}r_{0}=\frac{1}{2}\left(M_{i}-M_{0}-r_{i,0}^{2}\right).$$

When the number of UAVs is n=3, the system of (A3) can be solved using the elimination method to express *x* and *y*:

(A4) $$\left\{\begin{array}{cc} x & =P_{1}+Q_{1}r_{0}\\ y & =P_{2}+Q_{2}r_{0} \end{array},\right.$$

where $P_{1},P_{2},Q_{1},$ and $Q_{2}$ are the coefficients derived from (A3) using the elimination method. By setting i=0 and squaring both sides of (1), we obtain

(A5) $$r_{0}^{2}=M_{0}-2x_{0}x-2y_{0}y+x^{2}+y^{2}.$$

Substituting (A3) into (A5) obtains

(A6) $$Ar_{0}^{2}+Br_{0}+C=0,$$

where A,B, and *C* are the coefficients of the quadratic equation in terms of $r_{0}$ obtained by substituting *x* and *y* from (A4) into (A5). This equation is a quadratic equation in terms of $r_{0}$. Solving this equation and retaining the positive solution provide the viable value for $r_{0}$. If two positive solutions are obtained, the unreasonable solution should be excluded based on practical conditions and prior information. The positive solution of $r_{0}$ is then substituted back into (A4) to determine the coordinates of the target location.

When the number of UAVs is n≥4, the number of TDOA measurements exceeds the number of unknown variables. In this scenario, a two-step WLS estimation can be employed to fully utilize redundant data and achieve a more accurate localization estimate. At this stage, $r_{0}$ in (A5) is a nonlinear term related to the target location. We treat it as an unknown variable that is included in

(A7) $$Z=\left[\begin{array}{c} x\\ y\\ r_{0} \end{array}\right].$$

Let $Z^{0}$ represent the true values of Z corresponding to the true target location. We then establish the linear equation with TDOA noise

(A8) $$\psi =h-GZ^{0},$$

where ψ is the error vector caused by TDOA measurement noise, and

(A9) $$h=\frac{1}{2}\left[\begin{array}{c} M_{1}-M_{0}-r_{1,0}^{2}\\ M_{2}-M_{0}-r_{2,0}^{2}\\ \vdots \\ M_{n}-M_{0}-r_{n-1,0}^{2} \end{array}\right],$$

(A10) $$G=\left[\begin{array}{ccc} x_{1,0} & y_{1,0} & r_{1,0}\\ x_{2,0} & y_{2,0} & r_{2,0}\\ \vdots & \vdots & \vdots \\ x_{n-1,0} & y_{n-1,0} & r_{n-1,0} \end{array}\right].$$

The covariance matrix Ψ of the error vector ψ is

(A11) $$\Psi =E[\psi \psi^{T}]=BQB,$$

where

(A12) $$B=\mathrm{diag}\{r_{1},r_{2},\ldots r_{n-1}\}.$$

Q is the covariance matrix of the measurements.

(A13) $$Q=\mathrm{diag}\{\sigma_{1,0}^{2},\sigma_{2,0}^{2},\ldots ,\sigma_{n-1,0}^{2}\}.$$

Assuming that the elements of the error vector Z are mutually independent, the covariance matrix Ψ can be approximated by the matrix Q. Using the WLS method, the maximum likelihood estimate of Z can be expressed as

(A14) $$Z=\mathrm{argmin}\left\{{\left(h-GZ\right)}^{T}\Psi^{-1}\left(h-GZ\right)\right\}\approx \left(G^{T}Q^{-1}G\right)G^{T}Q^{-1}h.$$

To achieve a more precise localization result, a second WLS estimation step is performed. At this point, we construct a noise vector $\psi^{\prime }$ similar to (A8)

(A15) $$\psi^{\prime }=h^{\prime }-G^{\prime }Z^{\prime },$$

where

(A16) $$h^{\prime }=\left[\begin{array}{c} {\left(Z_{1}-x_{0}\right)}^{2}\\ {\left(Z_{2}-y_{0}\right)}^{2}\\ Z_{3}^{2} \end{array}\right],$$

(A17) $$G^{\prime }=\left[\begin{array}{cc} 1 & 0\\ 0 & 1\\ 1 & 1 \end{array}\right],$$

(A18) $$Z^{\prime }=\left[\begin{array}{c} x-x_{0}\\ y-y_{0} \end{array}\right].\;$$

The covariance matrix of the error vector $\Psi^{\prime }$ is

(A19) $$\Psi^{\prime }=E\left[\psi^{\prime }{\psi^{\prime }}^{T}\right]=4B^{\prime }\mathrm{cov}\left(Z\right)B^{\prime },$$

where $B^{\prime }=\mathrm{diag}\left(Z_{1}-x_{1},Z_{2}-y_{1},Z_{3}\right)$. The WLS localization estimate can be obtained as follows

(A20) $$Z^{\prime }={\left({G^{\prime }}^{T}\Psi^{\prime }-1G^{\prime }\right)}^{-1}{G^{\prime }}^{T}\Psi^{\prime }-1h^{\prime },$$

The final estimated position of the target is

(A21) $$\left[\begin{array}{c} x\\ y \end{array}\right]=\pm \sqrt{Z^{\prime }}\left[\begin{array}{c} x_{0}\\ y_{0} \end{array}\right].$$
